# The Influence of Processing Using Conventional and Hybrid Methods on the Composition, Polysaccharide Profiles and Selected Properties of Wheat Flour Enriched with Baking Enzymes

**DOI:** 10.3390/foods13182957

**Published:** 2024-09-18

**Authors:** Piotr Lewko, Agnieszka Wójtowicz, Daniel M. Kamiński

**Affiliations:** 1 Department of Thermal Technology and Food Process Engineering, University of Life Sciences in Lublin, Głęboka 31, 20-612 Lublin, Poland; piotr.lewko@up.lublin.pl; 2 PZZ Lubella GMW Sp. z o. o., Wrotkowska 1, 20-469 Lublin, Poland; 3 Department of Crystallography, Maria Curie-Sklodowska University, Maria Curie-Skłodowska Sq. 2, 20-031 Lublin, Poland; daniel.kaminski@umcs.lublin.pl

**Keywords:** dry heating, hydrothermal treatment, extrusion cooking, wheat flour, bakery enzyme, arabinoxylans, rheological properties, structure

## Abstract

In this study, a developed wheat flour blend (F), consisting of a high content of non-starch polysaccharides, was fortified with cellulase (C) and a cellulase–xylanase complex (CX) and then processed via conventional and hybrid treatment methods. Dry heating (T), hydrothermal treatment (H) and extrusion processing (E) were applied without or with enzyme addition as hybrid treatments. Proximate composition and polysaccharide profiles selected techno-functional and structural properties of modified wheat flours, were analyzed. Conventional and hybrid treatments induced changes in polysaccharide fraction compositions (especially the arabinoxylans) and the rheology of modified flour. Dry heating caused an inconsiderable effect on flour composition but reduced its baking value, mainly by reducing the elasticity of the dough and worsening the strain hardening index, from 49.27% (F) to 44.83% (TF) and from 1.66 (F) to 1.48 (TF), respectively. The enzymes added improved the rheological properties and baking strength, enhancing the quality of gluten proteins. Hydrothermal enzyme-assisted treatment increased flour viscosity by 14–26% and improved the dough stability by 12–21%; however, the use of steam negatively affected the protein structure, weakening dough stretchiness and elasticity. Extrusion, especially enzyme-assisted, significantly increased the hydration properties by 55–67% but lowered dough stability, fat content and initial gelatinization temperature due to the changes in the starch, mostly induced by the hybrid enzymatic–extrusion treatment. The structure of extruded flours was different from that obtained for other treatments where the peak intensity at 20° was the highest, suggesting the presence of amorphous phases of amylose and lipids. The results can be helpful in the selection of processing conditions so as to obtain flour products with specific techno-functional properties.

## 1. Introduction

Dry heating, hydrothermal or pressure–thermal treatments are useful in modifying the physical, rheological, technological and functional features or storage stability of wheat flour and other cereal products [1,2,3]. Thermal treatment reduces the natural enzyme activity, limits the moisture content and changes lipid fractions present mostly in bran-rich flour, thus extending the shelf life of cereal flours and products [4]. Dry heating mostly affects gluten proteins and starch molecules. At temperatures above 50 °C, gluten proteins are straightened or form gluten aggregates, thus modifying dough strength. As reported in other research, the changes during heating between 55 °C and 95 °C are connected with variable conformational changes, particularly in the glutenin proteins, and compositions of protein fractions. Data presented by Wang et al. [5] indicate that there are heat-induced alterations in gluten proteins at temperatures above 55 °C, which appear to be involved in the loss of functionality (baking performance) on heating. Between 55 and 75 °C, chemical changes occur in the protein component, new disulfide bond formation is observed, and the glutenin fraction is affected predominantly, as reported by Schofield et al. [6]. Glutenin changes that occurred in the temperature range of 65–70 °C were observed because of cross-linking temperature. The glutenin proteins are unfolded upon heating up to 75 °C, and this facilitates sulfhydryl/disulfide bond rearrangement [5,7]. At temperatures above 75 °C, the gliadin proteins are also affected, but the most important changes were observed around 80 °C due to protein polymer cross-linking. Within the temperature limit of 95 °C, gliadin binds with glutenin through non-covalent interactions and thus forms a viscoelastic gluten protein network [6]. Treatment above 95 °C is far more drastic, as in HTST processing, which causes thermal denaturation and induces permanent modifications in conformation and association, affecting the protein structure and producing interchain disulfide binding [8].

In comparison to hydrothermal treatment, dry heat treatment does not require external water and can be stored directly without drying, making it convenient for industrial production operations [9]. Moreover, dry thermal treatment is less drastic than heating in the presence of steam, mostly by limiting access to additional water. The changing intensity in protein, starch and fiber taking place during hydrothermal treatment depends on the water content, the time–temperature profile and the type of treatment: heated rolls, autoclaving, steaming, atomization or extrusion [10]. In hydrothermal modifications, the pregelatinization of starch and the partial denaturation of proteins occur; hence, cereal flour or bran is characterized by increased viscosity post-treatment. These hydrothermally modified flours can be used to produce functional flours used as thickeners or binders in soups, sauces, coatings or baby food [4].

More intensive treatment is obtained by the extrusion-cooking technique, which combines the effect of temperature, pressure, shearing forces and residence time with varied water accessibility in processing. Wheat components, especially gluten, are significantly involved in the structural, rheological and textural formation of the extrudates, and the main effect of protein fragmentation or aggregation in the extrusion process is generated through intermolecular changes in the disulfide bonds [11]. Moreover, starch can be partly or completely gelatinized as influenced by the initial moisture in raw materials and moisturizing level, range of treatment temperature, intensity of shearing and configuration of the device. Extrusion cooking is considered an effective treatment for converting insoluble fibers into soluble fractions [12,13].

Enzyme addition is a common method for starch, flour or bran modification, as enzyme addition may modify fiber fraction compositions, dough development ability and stability or increase absorption properties of flours. The main enzymes useful in bakery are amylases to change starch into simple sugars and dextrins, oxidases to enhance dough strengthening and whitening, hemicellulases for improving the gluten strength, proteases for reducing its elasticity and lipases for elongating the shelf life. All enzymes execute significant roles in the formation of bread dough and pan volume, texture, color and browning reactions throughout the time of baking or retrogradation and staling reduction [14]. The addition of cellulase or xylanase has been reported to improve the functionality of non-starch polysaccharides, which are mainly found in the external layers of cereal grains [15].

Soluble and insoluble arabinoxylans (AXs) and other non-starch polysaccharides (NSPs) as fractions of total dietary fiber are found in wheat flour and bran [16]. Most arabinoxylans intertwined with other macromolecules or embedded in cell walls are difficult to extract with water, but selected processing conditions can increase or change the structure and solubility or extractability of AX and other NSP fractions [17,18]. Singh et al. [19] found extrusion suitable for increasing the proportion of extractable dietary fiber, including AXs. AXs and NSPs are dietary fibers with variable properties depending on cereal variety, treatment method and internal composition. They are quite resistant to high-temperature treatments, due to contained hemicelluloses of varied degradation temperatures (243–279 °C for xylan and 332 °C for arabinoxylan, xyloglucan and β-glucan) [20]. Demuth et al. [21] reported significant structure changes in wheat bran water-soluble fractions of arabinoxylans after extrusion processing. Treatment of corn bran, as reported by Singkhornart et al. [22], reduced the content of sugars and soluble arabinoxylans after extrusion depending on the application or not of some chemical pretreatment or the application of various moisture levels or screw speed during modification. Several researchers have modified cereal products using coupled thermal/extrusion processing with enzyme applications, especially in bran fractions. Kong et al. [12], for example, tested coupled enzymatic–extrusion modification of bran from black wheat, by application of cellulase, high-temperature α-amylase, acid protease and xylanase alone or in combination to determine the effect upon water extractable arabinoxylan (WEAX). WEAX content and cholesterol adsorption capacity as well as oil and water retention ability increased dramatically, most likely as a result of the effect of loose porous structures after treatment.

The aim of this study was to assess the application of conventional dry thermal heating, hydrothermal treatment and low-temperature extrusion treatment in modifying developed wheat flour properties whether as individual or hybrid treatments with cellulase or cellulase–xylanase enzyme additions. In the work, chemical, rheological and structural properties were determined, with the hypothesis being that hybrid treatment more greatly affects the polysaccharide compositions, hydration capacity and techno-functional properties of modified developed wheat flour than does individual enzymatic, thermal, hydrothermal or extrusion treatment.

## 2. Materials and Methods

### 2.1. Materials

A newly developed flour blend (F) from common wheat of the Laudis variety was used for the tests. This consisted of flours of high arabinoxylan amounts from selected breaking, milling, reducing, sifting and sorting passages developed and composed by Lewko et al. [23], characterized by the wet gluten content of 31% (tested according to the ICC 155 method [24] by using a Perten Glutomatic 2200) (PerkinElmer Inc., Waltham, MA, USA) and a falling number of 340 s (tested according to ICC 107/1 standard method) [24].

Commercial baking enzymes were added to the flour as powders: Bakezyme^®^ WholeGain—cellulase from *Trichoderma reesei* (DSM Food Specialties B.V., Delft, The Netherlands) with declared enzyme activity 1475 EGU/g (±5%); and VERON 292—xylanase from *Aspergillus niger* (AB Enzymes GmbH, Darmstadt, Germany) with declared enzyme activity min 1701 XylH/g. Cellulase enzymes were employed for wheat flour fortification in an amount of 120 ppm (samples marked with C), and a complex of cellulase and xylanase enzymes was used for flour fortification in amounts of 60 ppm and 50 ppm, respectively (samples marked CX). The amount of the enzymes was determined based on preliminary tests and on the recommendations of the enzyme manufacturers.

### 2.2. Enzymatic Hydrolysis

**Figure 1:**
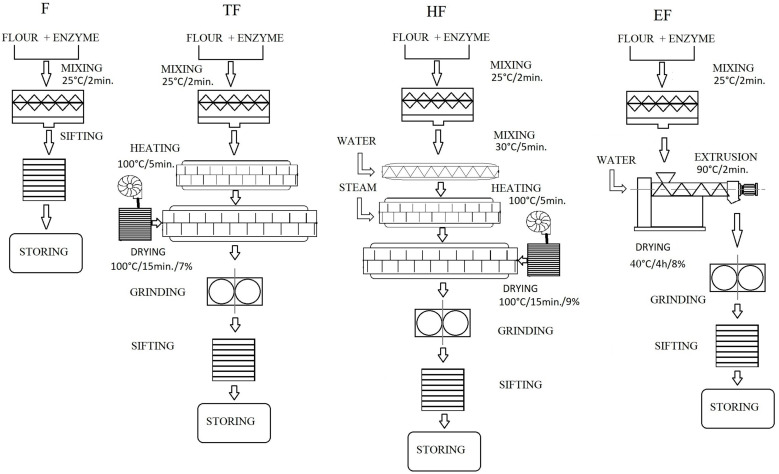
Schematic diagram of treatment methods.

Wheat flour (F) at 14% moisture content was prepared with 120 ppm of powdered cellulase (FC) and a combined 50 + 60 ppm of cellulase–xylanase complex (FCX). Components were mixed for 2 min at room temperature by using a laboratory ribbon mixer (Konstal—Zakład Mechaniczny CNC Zbigniew Własiuk, Lublin, Poland), left for resting for 2 h before measurements and sifted to uniform particle size below 300 µm (Figure 1).

### 2.3. Dry Heat Treatment

Dry thermal treatment (T) was carried out for wheat flour (TF) and flour fortified with enzymes (TFC and TFCX) by using an installation for thermal treatment owned by PZZ Lubella, Lublin, Poland (prototype installation). A schematic diagram of treatment runs is presented in Figure 1 with applied conditions. During the 5 min dry heat treatment, mixed samples at 14% moisture content were heated inside the barrel with a rotating screw, where the heating jacket temperature was set to 100 °C, with the product temperature measured during the tests to not exceed 50 °C. The prototype installation used for the tests was equipped with sensors located inside the barrel with a rotating screw. The temperature in the barrel was regulated by the temperature of the oil in the heating jacket of the device and the efficiency of the supplied amount of flour. After reaching the required processing temperature, the heating jacket was stopped and the flour was removed immediately by a two-way distributor; if the parameters were exceeded or were too low, the flour was directed to a separate tank, and the flour processed in the assumed conditions was collected as a test sample for further tests and sent for drying. The efficiency of this procedure is 650 kg of product/h. After dry heating or hybrid heat–enzymatic treatment, the flour was dried in an air dryer at 100 °C with an oil heating jacket to stop enzyme activity and to obtain a final moisture of 7%. To check the final moisture content, a fast method was employed using NIR Perten DA7250 (PerkinElmer Inc., Waltham, MA, USA). The samples were ground and sieved using a square sifter (Toruńskie Zakłady Urządzeń Młyńskich Spomasz S.A., Toruń, Poland) to homogenize the material, remove aggregates and obtain particle sizes below 300 µm, and 200 kg was collected for further research.

### 2.4. Hydrothermal Treatment

During the hydrothermal modification (H), the base wheat flour (HF) and flour fortified with enzymes (HFC and HFCX), prepared according to the procedure shown in Figure 1, were processed in a prototype installation (owned by PZZ Lubella). Flour samples mixed for 2 min with enzymes were transferred to a single-screw pre-conditioner at 30 °C with 20 L/h of water and additionally heated with steam injection for 5 min at a set heating jacket temperature of 100 °C, to reach a product temperature measured during the tests not exceeding 65 °C. The prototype installation used for the tests was equipped with sensors located inside the barrel with a rotating screw. The temperature in the barrel was regulated by the temperature of the oil in the heating jacket of the device and the efficiency of the supplied amount of flour. After reaching the required processing temperature, the heating jacket was stopped and the flour was removed immediately by a two-way distributor; if the parameters were exceeded or were too low, the flour was directed to a separate tank, and the flour processed in the assumed conditions was collected as a test sample for further tests and sent for drying. The obtained flour was then dried in an air dryer at 100 °C with an oil heating jacket to stop enzyme activity and to obtain a final moisture of 9%. To check the final moisture content, a fast method was employed using NIR Perten DA7250 (PerkinElmer Inc., Waltham, MA, USA). The efficiency of prototype installation with steam injection is 650 kg/h. The samples were ground and sieved using a square sifter (Toruńskie Zakłady Urządzeń Młyńskich Spomasz S.A., Toruń, Poland) to homogenize the material, remove aggregates and obtain particle sizes below 300 µm. A total of 200 kg of hydrothermally or hybrid-modified flour was gathered for subsequent tests.

### 2.5. Low-Temperature Extrusion-Cooking Treatment

Tests on the extrusion processing (E) of flour without (EF) and with various enzyme additions (EFC and EFCX) were carried out using an Evolum25 co-rotating twin-screw extruder (Clextral, Firminy, France) with an L/D = 24 configuration, with screws of 25 mm in diameter and with a single open die with a diameter of 3 mm. The feeding rate of dry components was set at 10 kg/h by using a volumetric gravity feeder (Brabender^®^ GmbH & Co. KG, Duisburg, Germany). The tests were performed at the feed moisture level of 27% by adding the water via a water pump directly into the second section of the extruder working at 400 rpm rotational screws speed. During the monitored low-temperature extrusion process, the temperatures in individual sections of the extruder were set from 40 °C in the dosing section and 50, 60, 65, 70 and 80 °C in the subsequent sections, up to 85 °C at the die, and were stabilized by a heating/cooling jacket system. The total residence time of treatment inside the extruder did not exceed 2 min (Figure 1). Obtained samples were shredded via a cutting knife and dried at 40 °C in a laboratory shelf dryer to ensure storage moisture below 8%, which was checked by a rapid moisture analyzer MA50R.WH (Radwag Wagi Elektroniczne, Radom, Poland). Extrudates were ground in a TestChem laboratory grinder (Radlin, Poland) and sifted to obtain a similar particle size below 300 µm, and samples were taken for further tests.

### 2.6. Proximate Composition Analysis

The selected chemical characteristics of native and modified flours were determined according to standard methods: AACC 46–10 method for protein (Nx6.25), AACC 30–10 method for fat and AACC 08–01 method for ash [25]. The 991.43 method was used to evaluate soluble (SDF) and insoluble (IDF) fractions and the content of total dietary fiber (TDF) [26,27]. Moisture content was evaluated in accordance with the ICC 110/1 method [24].

### 2.7. Content and Fractions of Non-Starch Polysaccharides and Arabinoxylan Analysis

The content of non-starch polysaccharides (NSPs) was determined by gas chromatography according to Englyst and Cummings [28]. The total NSP (T-NSP) content, soluble (S-NSP) and insoluble (I-NSP) fractions, as well as the content of total arabinoxylans (T-AX), insoluble (I-AX) and soluble (S-AX) fractions, were ascertained according to the procedure described by Fraś [29]. After acid hydrolysis of soluble and insoluble fractions, monosaccharides were detected in each fraction. The obtained hydrolysates were converted into volatile alditol acetates. To each sample (1 mL), 2 drops of 2-octanol, 0.26–0.28 mL of 12 M ammonia solution and 0.1 mL of sodium borohydride solution in ammonia (100 mg BH_4_ in 1 mL of 3M NH_4_OH) were added. After 40 min of incubation at 40 °C, 0.1 mL of glacial acetic acid was added to the hydrolysate and mixed, and then 0.2 mL of 1-methylimidazole and 2 mL of acetic anhydride were added to 0.2 mL of the collected sample. The prepared solution was cooled for 30 min and then 4 mL of distilled water and 1.15 mL of dichloromethane were added and shaken for 1 min. The aqueous phase was removed, and the organic phase was analyzed on an Autosystem XL gas chromatograph from Perkin Elmer (Shelton, CT, USA), equipped with an autosampler, a split injector, a flame ionization detector (FID) and an Rtx-225 capillary quartz column (0.53 mm × 30 m). Chromatograph operating parameters included the following: carrier gas helium, flow 2 mL/min, injector temperature 275 °C and detector temperature 275 °C. For the column temperature program, parameters included the following: initial temperature 185 °C, 1 min; increase 5 °C/min to 215 °C; and isotherm 215 °C, 10 min [29]. Gas chromatography allowed for the identification of the soluble and insoluble fractions of individual sugars: arabinose, xylose, mannose, galactose and glucose.

### 2.8. Hydration and Retention Properties

Solvent Retention Capacity (SRC) tests of native and modified flours were carried out in accordance with the AACC 56-11.02 approved procedure [25]. SRC was calculated as the retained solvent mass after centrifugation of the swollen flour and expressed as a percentage of the dry flour mass (amended to 14% moisture). Several solvent types were used: deionized water (WaSRC), 50 wt% sucrose-in-water solution (SuSRC), 5 wt% lactic acid-in-water solution (LaSRC) and 5 wt% sodium carbonate-in-water solution (ScSRC). A total of 5 g of samples placed in a 50 mL centrifuge tube was mingled with 25 g of solvents. Samples were then allowed to stand for 20 min and stirred every 5 min for 5 s to solvate. Subsequently, samples underwent centrifugation at 2500 rpm for 15 min, and the filtrate was poured off and allowed to stand for 10 min. SRC was calculated after weighting the samples. Calculation of the gluten performance index (GPI) included a division of LaSRC results by consolidating the results of SuSRC and ScSRC [30].

### 2.9. Pasting Properties

Pasting properties according to the ICC 169 procedure [24] were evaluated on a Brabender Viscograph-E (Brabender GmbH & Co., Duisburg, Germany) working with 75 rpm and 700 cmg torque. In total, 80 g of flour (adjusted to 14% moisture content) and 450 mL of distilled water were mixed, the prepared slurry was placed in the heating chamber, and the spindle was attached. The heating/cooling profile was as follows: heating from 30 °C to 93 °C at a rate of 1.5 °C/min, holding at 93 °C for 15 min, cooling to 50 °C at a rate of 3 °C/min and finally, holding at 55 °C for 15 min. Viscosity (mPas) was recorded as a resistance to stirring. The following pasting characteristics were obtained via Viscograph-E software (version 4.1.1): maximum viscosity, trough viscosity, final viscosity, breakdown (max viscosity minus trough viscosity) and setback (final viscosity minus trough viscosity), as well as the beginning and end of gelatinization temperatures.

### 2.10. Rheological Tests

Rheological properties were evaluated using the listed instruments: Mixolab (Chopin Technologies, Villeneuve-La-Garenne, France) in accordance with the ICC 173 method, Brabender Farinograph-E apparatus (Brabender, Duisburg, Germany) in accordance with the ICC 115/1 method and Alveograph (Chopin Technologies, Villeneuve-La-Garenne, France) in accordance with the ICC 121 method [24].

Rheological properties by Mixolab were determined based on the Chopin+ flour protocol with the following settings: mixing speed—80 rpm; total analysis time—45 min; dough weight—75 g; and hydration water temperature—30 °C. Flour and water were added accordingly to obtain a dough maximum consistency of 1.10 Nm (±0.05). The test was performed using a standard protocol: 8 min at 30 °C, heating for 15 min at a rate of 4 °C/min, holding for 7 min at 90 °C, cooling down to 50 °C for 10 min at a speed of 4 °C/min and finally, holding for 5 min at 50 °C [31]. Several properties have been assessed: water absorption (Hyd), protein weakening (C2), starch gelatinization (C3), amylase activity (C4) and starch retrogradation (C5) [32].

Rheological properties were tested by the Farinograph working with a standardized protocol [33]. Water absorption (WA) was expressed as the % of the water required to obtain a dough with a consistency of 500 BU or corrected at 14%, and dough development time (DT) was observed as the time to attain a consistency of 500 BU); dough stability (S), degree of softening (DoS and DoS12 after 12 min) and quality number (QN) were recorded.

Alveograph working with a standard procedure was used to determine the following characteristics: baking strength (W) calculated from the surface area under the curve, dough strength (P) calculated as the maximum pressure required to form the dough bubble expressing dough resistance, extensibility (L) of the dough as the length of the curve, elasticity index (Ie) [34], strain hardening index (SH) and P/L as a configuration ratio [35].

### 2.11. X-ray Diffraction Analysis

The ground flour samples were subjected to X-ray diffraction (XRD) using a high-resolution Empyrean powder diffractometer (PANalytical, The Netherlands) with Cu Kα1 radiation (λ = 1.54178 Å). Samples were measured in θ–2θ geometry, over an angle range from 10 to 70°, with a step size of 0.01° and a counting time of 400 s per data point. All measurements were carried out at room temperature and an RH of 28% [36].

### 2.12. Microstructure Observations

Flour microstructure was observed with a scanning electron microscope Vega Tescan LMU (Tescan, Brno, Czech Republic) at an accelerating voltage of 20 keV. Powdered samples were mounted on aluminum specimen stubs with double-sided adhesive silver tape and sprayed with gold using Sputter Coater Emitech K550X (Emitech, Essex, UK). SME pictures were taken with magnifications of 600× and 2000× [37].

### 2.13. Statistical Analysis

All analyses were performed in triplicate. One-way ANOVA was performed using Statistica 13.3 software (StatSoft, Inc., Tulsa, OK, USA), followed by Tukey’s least significant difference (LSD) post hoc test to compare means at the 0.05 significance level. Pearson’s correlation coefficients were determined to find the correlations between variables using Statistica 13.3 software (StatSoft, Inc., Tulsa, OK, USA) within the 95% confidence interval.

## 3. Results and Discussion

### 3.1. Effect of Treatment Method on Proximate Composition

Basic chemical components in the developed wheat flour varied depending on treatment methods. The moisture content of native flour without or with enzymes was similar around 14%. Processed flours exhibited lower moisture content due to the drying after treatment and values were from 7.01% to 7.23% for dry thermal treatments, from 8.98% to 9.28% for hydrothermal treatments and from 8.06% to 8.26% for extruded flours, with the unessential effect of enzymes added (Table 1). These moisture values were also used during testing rheological properties as a base for specific calculations. The results of chemical components are presented as is and were slightly dependent on the flour moisture content. The content of protein in native wheat flour was 14.62%, and the application of enzymes did not significantly affect protein content (Table 1). Application of dry heating, hydrothermal treatment and extrusion, both as individual or hybrid treatments with the addition of enzymes, resulted in lower moisture of flour, which had an effect on the protein content results presented in Table 1 as expressed in wet mass. These results showed increased protein content in treated samples, but, as expressed in dry mass, the protein content decreased in all treated samples. As a result of processing at high temperatures (above 50 °C), changes occur in the molecular conformation of the protein, such as the unfolding of gluten proteins, the formation of gluten aggregates, with reduced extractability, changes between sulfhydryl/disulfide bond exchange reactions leading to glutenin polymerization and modified molecular mass distribution, which may affect the final protein content and the possibility of forming unextractable polymeric proteins (UPPs), especially in the presence of starch, as confirmed by Guerrieri and Cerletti [8] based on internal fluorescence conformational changes in proteins, Hu et al. [33] on CSLM images and Schofield et al. [6] by chromatographic analysis. Differences between samples were very small in proximate composition, but it was worth noting that when hybrid treatment with CX complex addition occurred, irrespective of the treatment method, the protein content was higher in all modified flours with CX, both as expressed in wet and dry mass. The probable mechanism of the CX enzyme complex action, which was used to improve gluten quality, is the destruction of cellulose fibers by cellulase and the limitation of water absorption by water-unextractable arabinoxylans by xylanases, thus improving gluten hydration [38]. A significant lowering of extractable fat content was observed in samples undergoing extrusion modification both without/with enzyme addition (EF, EFC and EFCX samples). Alam et al. [39] stated that fat is able to form complexes with starch or protein during extrusion cooking, and the obtained results confirm this observation. The fat content was at least 5-fold lower than in native flour and thermally treated samples. Enzyme addition had an indistinct effect on fat content, especially in F, FC and FCX, as well as in TF-, TFC- and TFCX-modified flours. In samples processed with hydrothermal treatment, both C and CX enzyme additions decreased fat content from 1.37% in HF to 1.30% in HFC and HFCX samples. Application of modification methods slightly increased ash content in all samples, as compared to untreated and enzymatic fortified flour.

The greatest differences were observed in dietary fiber content and its fractions. Application of C and a CX complex under environmental conditions lowered TDF content in FC and FCX samples, with an 18–19% increased ratio of insoluble/soluble fractions of dietary fiber (Table 1). TDF increased significantly after dry thermal treatment without (TF) and with added enzymes (TFC and TFCX), but the IDF/SDF ratio was lower after the hybrid method application. Individual hydrothermal treatments slightly increased the TDF content, with increasing soluble fraction content in the HF samples, as compared to the native sample. Hybrid HFC and EFC treatments showed increased content of TDF due to cellulase action on fibrous components present in flour under pressure–thermal conditions, hence the release of more insoluble fiber fractions. In contrast, CX addition significantly lowered TDF content in HFCX and EFCX samples. The synergic effect of both enzymes also significantly lowered the detection possibility of fibrous fractions—probably due to their enzymatic hydrolysis being supported by the integrated water temperature effect. A significant increase in SDF was observed in HF and in extruded samples which generated similar or lower values of the IDF/SDF ratio than in native F wheat flour (Table 1).

The impact of extrusion conditions as extruder barrel temperature or screw speed, on different dietary fiber components, may vary depending on processing conditions. Alam et al. [39], for example, reported a significant increase in SDF and TDF fractions in extruded rye bran when both in-barrel water feeding and pre-conditioning were applied during twin-screw extrusion at 130 °C. A decrease in TDF may also be observed due to the degradation of insoluble parts into smaller-molecular-weight compounds by extrusion. Lee et al. [40] tested the effect of various cooking methods on wheat bran and reported extrusion to be the most effective in increasing the TPC, the SDF content, the bulk density of bran and the midline peak time.

### 3.2. Influence of Modification Conditions on Polysaccharide Fractions

Consumption of AX, the dominant dietary fiber component in bran, helps to reduce glucose and insulin levels in food. Table 2 and Table 3 present the effect of the modification method on NSP and AX compositions of insoluble and soluble fractions in treated wheat flour without/with enzyme addition. Table 4 reveals summarized NSP and AX total insoluble and soluble fractions depending on the treatment method.

Regarding the insoluble sugars present in native and modified flour, after applying dry thermal treatment TF, the samples demonstrated increasing content of insoluble fractions of mannose, galactose, glucose, arabinose and xylose, as compared to native F flour (Table 2). However, enzyme treatment significantly lowered insoluble glucose, arabinose and xylose fractions in FC and FCX samples, as compared to native samples. Moreover, enzyme addition in TFC and TFCX hybrid treatment, with the exception of I-mannose, lowered insoluble sugar content, while HF and hybrid HFC and HFCX methods formed more insoluble fractions of mannose, galactose and glucose, whereas I-arabinose levels lowered significantly. After extrusion and hybrid treatments, insoluble arabinose and xylose were lowered most significantly. Similar to the composition of soluble fractions of NSP and AX (Table 3), cellulase and cellulase–xylanase complex incorporation increased the soluble fraction contents of almost all sugars, suggesting the formation of more soluble fractions after enzymatic activity.

Dry thermal treatment lowered soluble mannose and xylose content, and enzyme additions showed differential effects on TFC and TFCX samples. HF treatment without enzymes demonstrated increased soluble sugar content compared to native F flour. C or CX complex addition lowered soluble mannose, but other sugar contents increased (such as S-glucose and S-arabinose) or seemed similar to native flour. The most significant differences were observed if extrusion or hybrid enzymatic–extrusion treatment was applied. Accordingly, an especially strong increase in soluble arabinose and xylose was noted, while the S-A/X ratio was the lowest. In general, modification methods, except for TF, slightly lowered the amount of total arabinoxylan content compared to native F flour, probably due to partial hydrolysis by enzymes or the formation of complexes.

A significant decrease in insoluble AX and a simultaneous increase in soluble AX were observed in extruded samples, without/with enzymes (Table 4). In almost all treated and hybrid-treated samples, the content of T-NSP decreased except for TF and all hydrothermally treated samples (HF, HFC and HFCX), as compared to native F. In contrast, soluble NSP fractions increased in FCX samples, and in HF and HFCX, as well as in EFC- and EFCX-treated flours.

In general, modification methods, except for TF, slightly lowered the amount of total arabinoxylan content compared to native F flour, probably due to partial hydrolysis by enzymes or the formation of complexes. Positive correlations (significant at *p* < 0.05) were noted in treated flours between I-arabinose (0.610), I-xylose (0.626), I-AX (0.639), I-NSP (0.682) and T-NSP (0.634) with insoluble fiber content. Soluble fiber fractions present in modified flour were also correlated positively with T-NSP at 0.633, and total fiber content was correlated with I-AX (0.606), I-NSP (0.661) and T-NSP (0.698). These correlations were not extremely high but were significant at *p* < 0.05. Andersson et al. [17] reported an increase in extractable dietary fiber, including AX and its soluble fractions, through extrusion processing. The applied high shearing forces cause fiber length reduction; therefore, it probably increases the accessible surface area for enzymatic hydrolysis. According to Yağcı et al. [41], extrusion experiments conducted at maximum barrel temperatures of 40, 75 and 110 °C enabled minimal degradation of bulgur bran hemicellulose. They also observed a significant reduction in glucose content after extrusion pretreatment, but an increase in hemicellulose, xylan and arabinose contents after combined alkali–extrusion treatments of bran. Corn fiber pretreated in a twin-screw extruder with different chemicals (NaOH and H_2_SO_4_ solutions) showed that increasing screw speed improved reducing sugar, soluble arabinoxylan content and the yield of corn fiber gum [22].

### 3.3. Effect of Treatment on Hydration and Retention Properties

Preferred techno-functional properties depend on flour designation in the bakery industry. For example, bread flour requires high water absorption, good gluten strength and relatively high damaged starch and arabinoxylan content, while cookie flour requires low water absorption, minimal gluten strength and low damaged starch and arabinoxylan content [42]. The absorption and retention capacity of flour or dough components may indicate allusive main components responsible for flour quality. The SRC method with no additional shearing and heating of components with La is useful for indicating the protein quality; Sc reveals starch quality and Su uncovers polysaccharide contents (especially pentosane structures). The results of SRC measurements of untreated, individual and hybrid method treatments for wheat flour are presented in Table 5.

Native F flour demonstrated 70.005% SRCWa, 114.973% SRCSu, 118.294% SRCLa and 87.839% SRCSc. Enzymatic action increased the Wa, Su, La and Sc retention capacity of FC and FCX flours, whereas TF treatment without enzymes slightly decreased the absorption and retention capacity, and when cellulase was used in the hybrid TFC flour, water hydration decreased. Moreover, La and Sc solutions in TFCX samples brought about opposite trends, and a slight increase in Su absorption was noted in both TFC and TFCX samples, indicating increased activity of fibrous fractions under dry heating. Furthermore, hydrothermal treatments without/with enzymes lowered SRC values, with the most significant decrease in SRCLa, suggesting limited absorption by proteins present in HF-, HFC- and HFCX-modified flours due to the possible formation of unextractable polymeric proteins (UPPs), especially in the presence of starch, after treatment above 50 °C, as reported by Guerrieri and Cerletti [8], Hu et al. [33] and Schofield et al. [6]. Van Steertegem et al. [43] reported decreased SRCLa values of commercial wheat flour subjected to either 2 or 5 h heating at 80 or 100 °C, indicating that heat treatment restricted the swelling ability of the protein network, which was related to protein cross-linking within the flour particles. Longer and more severe heat treatments indicated more cross-linking, leading to lower LaSRC, but when heat treatment was relatively mild, increases in WaSRC and SuSRC were observed. Moreover, they found decreases in both SDSEP (proteins extractable in sodium dodecyl sulfate (SDS)-containing medium) and free SH groups as a result of heating, indicating that the gluten proteins formed covalent disulfide (SS) cross-links and hence polymerized after treatment, thereby increasing gluten protein polymer size values and thus preventing the gluten proteins from swelling in the La-containing media [43]. Similar results were noted in our results for HF samples and this effect was limited after treatments with enzymes added.

Extrusion and hybrid enzymatic–extrusion treatments generated increased absorption ability and retention capacity in modified EF, EFC and EFCX flour, probably due to significant changes in proteins, starch and fiber because of the treatment intensity. Combined EFCX treatment strongly affected SRC due to having the highest values of retention capacity of all applied solvents (Table 5). This again reveals the intensity of extrusion and hybrid enzymatic–extrusion conditions on the tested wheat flour. Additionally, after any form of extrusion treatment, compared to other treatments, GPI values were the lowest. GPI, considering the overall effect of protein, starch and fibrous fractions, indicates the overall performance of glutenin capability in simulating dough behavior. Other treatments showed negligible or slight decreasing effects on GPI values, and only the FCX method increased GPI. GPI results were significantly (at *p* < 0.05) correlated in a positive trend with insoluble fiber content in modified flours with a coefficient of 0.628, with dough stability S (0.925), with rheological features C3 (0.955), C4 (0.928) and C5 (0.921), but negatively with Hyd (−0.968).

A negative correlation was found between I-xylose with SRCWa (−0.607) and SRCLa (−0.620). So, with an increased level of insoluble fractions, the I-NSP values of SRC decreased for all solvents used, and significant (*p* < 0.05) negative correlation coefficients were noted between I-NSP and SRCWa (−0.659), SRCSu (−0.670), SRCLa (−0.696) and SRCSc (−0.642). Moreover, a significant (at *p* < 0.05) correlation was found between T-NSP and SRCSu (−0.613). For certain soluble fractions of simple sugars, only significant positive correlations at *p* < 0.05 were found as significant between S-xylose and SRCWa (0.619), SRCLa (0.610) and SRCSc (0.601), which means that if S-xylose was higher, the flour absorption ability would be improved with the most visible action of gluten explained by SRCLa values. Also, hydration (Hyd) properties of modified flours were significantly (at *p* < 0.05) positively correlated with SRCWa values (0.992), SCRSu (0.970), SRC La (0.978) and SRCSc (0.995). Strong significant negative correlations at *p* < 0.05 were found between S, C3, C4, C5 and all SRC results for all solvents used, and correlation coefficients varied from −0.947 to −0.983. So, it can be stated that the hydration properties of modified flours are strongly related to rheological features related to starch changes after treatments.

Keppler et al. [44] tested the dry heat treatment of soft wheat flour by heating a thin layer at temperatures between 110 °C and 200 °C for between 1 and 30 min. They noted improved swelling ability and increased interactions of flour polymers (in particular arabinoxylans) of heat-treated flour at ambient conditions as tested by SRC tests. An increase in individual solvent (La, SC and Su) retention capacity was observed when flour was heated at an elongated time, and a decrease in GPI was significant at prolonged heating time. They connected the SRC profile with the impact of thermal treatment on the arabinoxylan fraction of flour, showing increased swelling behavior in the SRC test mostly upon heat.

Ma et al. [9] investigated the effects of wheat bran pretreatments by autoclaving, roasting, jet cooking, extrusion, puffing and high-temperature high-pressure cooking on steamed bread and pancake properties. All the pretreatments of bran imparted negative influences on the gluten index of whole wheat flour. These treatments significantly increased the water absorption index, water retention capacity and SDF content, but differently affected the microstructure, the median particle size, the bulk density of wheat bran and the dough mixing properties. Extrusion of bran may improve the crumb structure quality, stress relaxation score and springiness in steam bread, and autoclaving of bran improved the moistness of pancakes [40].

### 3.4. Impact of Heat Treatment Processes on Pasting Properties

Pasting properties indicate changes in starchy components, e.g., partial or complete gelatinization by thermal or mechanical treatments. Peak and hot viscosity are the maximum and the lowest viscosity of the starch paste for heating, respectively, and they reveal binding water effects and starch granule swelling. The final viscosity reflects the stability of cooled starch paste. Breakdown and setback indicate the paste’s resistance to heat and shear and the paste’s retrogradation as a result of cooling [45]. The results of pasting characteristics of native, enzyme-supported and individual or hybrid-treated wheat flour are presented in Table 6.

The maximum viscosity of native wheat flour was 1564 mPas, and the application of enzymes lowered the maximum, trough and final viscosity of enzyme-fortified samples. In dry-thermal-modified samples, viscosity was significantly lower as compared with native flour, and the application of enzymes induced increased viscosity in the TFC and TFCX samples. Hydrothermal treatment was the most influencing method for increasing viscosity, and, in this case, hybrid HFC treatment produced the highest viscosity values. Moreover, breakdown and setback were the highest, with the same beginning gelatinization temperature. Here, in TF, HF and hybrid treatments, the effect of enzymes was opposite to that in the F and E methods. Extruded EF and hybrid EFC- and EFCX-treated flours showed lower maximum viscosity than native F, FC or FCX samples, but the trough and final viscosities were higher than in native or dry-thermal-modified flours. A significantly lower beginning gelatinization temperature was noted in all extruded samples, indicating that starch was partly melted and gelatinized. Applied treatments resulted in increased setback values in HFC, EF and EFC samples (Table 6), suggesting increased starch paste stability and gel hardness. Other treatments, especially when enzymes were applied as individual C, a CX complex or in hybrid dry thermal treatment, lowered setback values.

Bucsella et al. [4] confirm that the hydrothermal process increased peak viscosity and setback values, compared to untreated flours, while treated bread flour showed higher peak viscosity than treated cake flour. Similar observations were reported for increased temperature and retention time [46]. Therefore, hydrothermally treated starch granules are more rigid and resistant to quick heating due to altered swelling behavior, as stated by McCann et al. [47]. The presence of protein and starch in wheat flour differentiates pasting properties by competition of protein and starch for water during hydration. Pasting properties were measured with greater access to water oppositely to dough tested in Mixolab because of the altered diffusion of water into starch granules through the formation of a starch–protein matrix and starch gelatinization during the pasting procedure [48]. This behavior was not observed in dough because of less accessible water [4]. In our research, significant (at *p* < 0.05) correlations were observed between breakdown values and C3 (0.677), C4 (0.745) and C5 (0.734), as indicated mostly by starch transformations during heating. Additionally, a significant correlation at *p* < 0.05 between pasting properties and dough properties was similar to breakdown with dough stability (0.746) and C2 (0.714), as a result of protein weakening. Where the highest protein destruction occurred, the highest C2 level showed the highest max viscosity with a correlation coefficient of 0.826 at *p* < 0.05. For samples HFC and HFCX, the highest gel formation properties were observed as confirmed by the highest viscosity because of the high stiffness of dough as confirmed by high C2 values, which correlated with setback values (0.626). These observations may be the effect of the hybrid action of enzymes used with the high level of the insoluble fraction of I-NSP in HFC and the high level of S-NSP in HFCX-modified flour (Table 4). Breakdown was also significantly (at *p* < 0.05) negatively correlated (−0.693) with SRCSu responsible for arabinoxylan absorption. But these pasting analysis results were less connected with other modified flour properties than results obtained in the dough matrix tested by Mixolab. Hu et al. [33] reported that the application of superheated steam into wheat grains resulted in lower gelatinization temperature and higher peak viscosity with increased time and temperature of treatments up to 200 °C, upon which starch damage was observed. Peak and final viscosities, as well as breakdown and setback, decreased gradually with increased content of damaged starch due to better hydration, and weakening and breakdown of the gelatinized granules were observed [49]. Ma et al. [50] reported that superheated steam processing resulted in a rise in peak viscosity compared with native wheat flour; similar to our research, these changes were correlated with the denaturation protein barriers surrounding starch granules. Deng et al. [51] found higher viscosity of enzymatic–extrusion-treated rice bran when the screw rate was low at low moisture content, due to the higher specific mechanical energy input, which softened the fiber and created a loose and porous structure. Higher water content in raw material, higher gelatinization and a more rapid increase in viscosity at low temperatures were reported by Liu et al. [52] if rice starch was extruded with a single-screw extruder. The pasting profile of wheat flours studied by Román et al. [53] showed lower viscosity profiles of non-enzymatically treated and extruded flours, confirming that gelatinization occurred during thermal treatments, similarly shown in our research. Breakdown and setback values were also reduced. Both extruded and native samples with additional enzymatic treatment showed very low viscosity and flat pasting profiles with no peak viscosity as a result of the hydrolytic activity of the enzyme on the starch.

### 3.5. Heat Treatment Impact on Rheological Dough Properties

In our study, water absorption tested by hydration measurement varied depending on the testing method. Hydration as tested via Mixolab corresponded very well (r = 0.900 at *p* < 0.05) to the water absorption determined by the Farinograph when corrected to 14% initial moisture. However, unlike the Farinograph, the Mixolab allowed for the assessment of the water absorption of modified flour subjected to the extrusion process. Native flour F demonstrated water absorption of 60.5%, while both thermal and hydrothermal treatment in the TF and HF samples reduced water absorption (Table 7). The addition of C and CX enzymes in all treated samples resulted in a slight increase in water absorption. For extrusion processing, as in the SRC study, a more than twofold increase in hydration was observed compared to other tested methods as a result of starch swelling or partial gelatinization during processing. The analyzed development time (DT) of dough from native F flour was short (1.92 min), but the addition of enzymes in the FC and FCX samples significantly enhanced this parameter. Dry thermal treatment resulted in a 2.5-fold increase in the DT without significant impact of enzyme use. Hydrothermal treatment elongated DT, especially in the HFC sample, to 7.40 min; this flour was characterized by high S-A/X (1.048) and the highest total fiber content (7.49%). In samples subjected to the extrusion treatment, DT did not differ significantly compared to unprocessed samples. Moreover, the stability of flour treated by dry heating was similar to F, while hydrothermal steam-assisted treatment resulted in increased stability, and low-temperature extrusion reduced stability by almost triple as compared to native flour with no significant effect of enzyme addition.

Mixolab measures the C2 parameter as a dough consistency loss during the exposure to physical–mechanical and thermal stress, and after heat-induced protein denaturation carbohydrate-dependent starch gelatinization (C3), amylase activity (C4) and starch gelling (C5) dominated. The results of rheological characteristics tested with the Mixolab procedure are presented in Table 7.

**Table 7:** Mixolab features of untreated and hybrid-treated flours.

Sample	Hyd (%)	DT (min)	S (min)	C2 (Nm)	C3 (Nm)	C4 (Nm)	C5 (Nm)
F	60.5 ± 0.1 ^b^	1.92 ± 0.19 ^a^	9.73 ± 0.12 ^e^	0.477 ± 0.01 ^c^	1.709 ± 0.01 ^c,d^	1.479 ± 0.01 ^e^	2.519 ± 0.00 ^e,f^
FC	60.6 ± 0.1 ^b^	2.66 ± 0.76 ^a,b^	9.60 ± 0.36 ^d,e^	0.432 ± 0.00 ^a,b^	1.673 ± 0.006 ^c^	1.433 ± 0.03 ^d,e^	2.414 ± 0.03 ^d,e^
FCX	61.2 ± 0.2 ^b^	3.22 ± 0.34 ^b^	9.37 ± 0.15 ^c,d,e^	0.423 ± 0.00 ^a^	1.674 ± 0.01 ^c^	1.351 ± 0.01 ^c^	2.173 ± 0.04 ^c^
TF	57.6 ± 0.3 ^a^	4.94 ± 0.39 ^c^	9.23 ± 0.06 ^b,c,d^	0.454 ± 0.01 ^b,c^	1.665 ± 0.01 ^c^	1.352 ± 0.02 ^c^	2.343 ± 0.07 ^d^
TFC	60.8 ± 0.2 ^b^	4.81 ± 0.20 ^c^	9.03 ± 0.15 ^b,c^	0.448 ± 0.01 ^a,b^	1.666 ± 0.01 ^c^	1.393 ± 0.01 ^c,d^	2.402 ± 0.06 ^d,e^
TFCX	61.6 ± 0.2 ^b^	4.68 ± 0.07 ^c^	8.83 ± 0.23 ^b^	0.441 ± 0.01 ^a,b^	1.657 ± 0.01 ^c^	1.382 ± 0.02 ^c,d^	2.330 ± 0.05 ^c,d^
HF	58.1 ± 0.4 ^a^	2.37 ± 0.38 ^a,b^	10.87 ± 0.23 ^f^	0.572 ± 0.01 ^e^	1.781 ± 0.01 ^d^	1.571 ± 0.05 ^f^	2.675 ± 0.13 ^f^
HFC	58.3 ± 0.2 ^a^	7.40 ± 0.94 ^d^	11.90 ± 0.10 ^g^	0.770 ± 0.00 ^g^	1.985 ± 0.00 ^e^	1.832 ± 0.01 ^g^	3.072 ± 0.02 ^g^
HFCX	58.7 ± 1.1 ^a^	3.14 ± 0.54 ^a,b^	11.77 ± 0.15 ^g^	0.699 ± 0.01 ^f^	1.939 ± 0.02 ^e^	1.801 ± 0.01 ^g^	2.952 ± 0.07 ^g^
EF	94.3 ± 1.5 ^c^	2.59 ± 0.01 ^a,b^	3.83 ± 0.06 ^a^	0.524 ± 0.02 ^d^	0.745 ± 0.02 ^b^	0.491 ± 0.02 ^b^	0.844 ± 0.03 ^b^
EFC	95.2 ± 0.1 ^c^	2.13 ± 0.20 ^a,b^	3.87 ± 0.06 ^a^	0.529 ± 0.00 ^d^	0.639 ± 0.10 ^a^	0.443 ± 0.00 ^b^	0.777 ± 0.01 ^a,b^
EFCX	101.2 ± 0.3 ^d^	2.66 ± 0.01 ^a,b^	3.42 ± 0.03 ^a^	0.566 ± 000 ^e^	0.576 ± 0.00 ^a^	0.366 ± 0.00 ^a^	0.657 ± 0.01 ^a,b^

F—flour; T—dry thermal treatment; H—hydrothermal treatment; E—extrusion treatment; C—cellulase enzyme; X–xylanase enzyme; Hyd—hydration capacity; DT—development time; S—stability C2—protein weakening; C3—starch gelatinization; C4—amylase activity; C5—starch retrogradation; ^a–g^—means indicated with similar letters in columns do not differ significantly at α = 0.05.

The C2 protein weakening parameter for TF samples did not differ from F, but a significant increase was observed for flours subjected to hydrothermal and extrusion processing. The highest C2 was observed in the HFC sample, and the effect was probably related to the highest fiber content, especially insoluble fractions, which resulted in the deterioration of the flour’s baking properties, as confirmed by alveographic analysis results presented later (in Table 9). Tested dough made with steam-assisted hydrothermally treated flour HF was characterized by high springiness and low elasticity caused by significant changes in the conformation of gluten proteins, preventing proper development of the gluten network or destruction of gluten. As described by Hong et al. [48], modification of wheat flour using superheated steam treatment causes the denaturation of proteins and the initial gelatinization of starch granules contained in wheat flour, thus reducing the access of water to the protein phase due to greater absorption by modified starch. The effects of these changes may be visible by the problem with the dough formation ability caused by the weakening of the gluten quality and a reduction in its elasticity. An increase in the C2 parameter compared to F was also observed for samples subjected to low-temperature extrusion. These changes were induced by the loss of the gluten–protein matrix properties due to high mechanical shearing. For C2, after heating to 60 °C, if the gluten is damaged, the C2 in HF is high, similar to what was reported by Lewko et al. [54] for low-temperature single-screw extrusion. For these tests, high elasticity P and low extensibility L were also noted (Table 9). The C3 and C4 parameters did not differ significantly in native flours without/with enzyme incorporation. The addition of enzymes to native flour reduced starch retrogradation level (C5) in FCX samples. Similarly, the thermal process did not affect the C3 value but slightly reduced the amylase activity of C4 and starch retrogradation (C5), without the significant influence of the enzymes. The highest differences in the impact of the processing on starch-related parameters after hydrothermal treatment were observed, wherein significant increases in C3, C4 and C5 (especially in the presence of C and the CX complex) were indicated. Some significant correlations were found between dough features tested with Mixolab and pasting properties, as reported in Section 3.4.

The extrusion process, in turn, significantly reduced the C3, C4 and C5 values, probably due to the high water absorption of flours obtained after this treatment. The above parameters had values inversely proportional to the flour’s water absorption (Hyd) with significant correlation coefficients at *p* < 0.05 of −0.980 with C3, −0.958 with C4, and −0.961 with C5, the lowest being for EFCX flour. Reduction in the gelling ability (C3) may be altered by the protein–pentosan–lipid complexes formed in extrusion processing. Bucsella et al. [4] reported that the C3 parameter at the heating phase is higher in cookie flour than in bread flour. In contrast, the gelling ability of both types of flour during the cooling phase (C5) is similar. Moreover, thermally treated flour shows higher C2 and lower weakening with similar DT of cake flour, but with prolonged stability. Minor differences between untreated and dry-treated flours, especially in C3 and C4 values, may be because of the insignificant effect of dry heating on starch structure and gelling properties. Bucsela et al. [4], comparing RVA and Mixolab, found that starch behaves differently in the dough matrix and differently in the suspension (difference in water content to flour), and flours were more degraded by the high process temperature of 96 °C in hydrothermal treatment. These changes were more similar to our results for flour samples treated using low-temperature extrusion—then, the described dependencies of rheological features can be confirmed which are consistent with the cited work [4]. Moreover, in our research, the applied parameters of hydrothermal treatment used in the prototype installation were less aggressive in order to better protect the enzyme used as additives from the effect of high temperature.

Farinographic assessment allows for predicting the baking quality and may indicate directions for the technological use of the tested products [33]. Table 8 shows the results of the Farinograph tests. Flours subjected to extrusion and hybrid enzymatic–extrusion processing could not be analyzed on a Farinograph due to the probable complete loss of wheat gluten functionality and the very high water absorption obtained.

The process of thermal heating of flour showed an insignificant impact on water absorption, especially if moisture was adjusted to 14%. Similar observations were made by Hu et al. [33] embracing thermal modification of wheat grains before milling, and Bucsella et al. [4] encompassing thermal and hydrothermal treatment on wheat flour for cakes and bread. In our study, steam-assisted hydrothermal treatment reduced the corrected water absorption of flour compared to F flour. In all treatments, the addition of enzymes resulted in a slight increase in water absorption, especially with CX, with the highest value being obtained for TFCX. Thermal processing of flour may create a more strengthened dough structure without changing its hydration properties [4]. Thermal treatment of flour also resulted in dough DT increase (the lowest for TF and the highest for TFCX). Hydrothermal treatment HF did not change the dough DT, as compared to native flour F. In the HFCX samples, DT was similar to samples subjected only to thermal treatment and was twice as long as in the HF test. The longest dough development time was observed for the HFC sample (18.5 min). These findings are in agreement with research presented by Hu et al. [33], who tested the superheated steam (SS) effect of flour and dough behavior. Dough development time and stability all showed an increasing trend with the extension of superheated steam processing time. After 4 min of processing, the development and stability times increased from NF of 1.2 min and 1.1 min to 8.5 min and 7.1 min for modified flour with superheated steam. The dough exhibited longer stability time and always showed a less weakening index. Dough development time reflected the resistance of the dough against the blades. Dough stability and the degree of softening gave an indication of dough strength and tenacity. The increase in development time and stability showed an enhanced resistance to successive mixing and an improved capacity to sustain shear stress. Combined with the lower degree of softening, it suggested that dough made of SS-treated flours was much stronger and tenacious than that made of NF and the effect of SS treatment on protein aggregation as well as protein–starch interaction [33]. HFC-treated flour was characterized by a high amount of T-NSP and a high content of I-AX and I-NSP fractions among the modified flours tested (Table 4). After adding only cellulase, the amount of soluble fractions of non-starch polysaccharides decreased and the amount of insoluble ones increased, while with the addition of a CX complex, an increase in the amount of soluble and a decrease in the amount of insoluble NSP fractions was noted. This may be the effect of the interaction of hydrothermal treatment in the presence of easily accessible water from steam injection combined with enzyme activity. Various cellulase activities, such as cellobiohydrolase and endoglucanase, can hydrolyze cellulose. The cellulase used in this study contained both a high-activity cellobiohydrolase polymer and an endoglucanase and was responsible for the breakdown of cellulose polymers. The main activity of cellobiohydrolase is opening the fibrils to xylanase action, which breaks down cell wall components, especially in the whole grain fraction. The final effect is increasing the amount of insoluble fractions and facilitating the action of other enzymes. So, the combination of cellulase and xylanase may improve the quality of the crumb because it breaks up cellulose fibers responsible for improved gluten stability and gas retention, without interfering with the action of xylanase, which hydrolyzes arabinoxylans to their soluble form [38].

P result elasticity from Alveograph tests decreased from 140 mm for HF to 97 mm in HFC, thereby decreasing W from 227 × 10^−4^ J in HF to 134 × 10^−4^ J in HFC (Table 9), which indicates that a large amount of insoluble NSP fractions influenced the quality of gluten proteins. These insoluble fractions influenced the result of long dough development time (DT) (18.5 min for HFC) and a small DoS of 7.7 BU for HFC because after using the enzyme cellulase and hydrothermal treatment processing of flour with a large amount of insoluble NSP, the dough made from this flour absorbed water very slowly and developed slowly, while the stiff dough was indicated by high C2 and the highest viscosity due to starch gelatinization, as confirmed by Viscograph tests. During Farinographic analysis, after obtaining optimal consistency, partially heated and gelatinized starch and insoluble fractions of polysaccharides competed for water, and the formed structure did not give the possibility of gluten development. In HFCX, a higher amount of soluble fractions improved gluten development ability (DT was 6.8 min), but still, in dough, the C2 value was high (0.699 Nm), with high springiness (P = 147 mm) and low extensibility (L = 31 mm), because denatured proteins after hydrothermal treatment were stiff and not elastic anymore.

Flour stability (S) was found to be dependent on the processing method used. When dry heated, stability decreased slightly to 11.5 min compared to F, but so did the degree of softening (DoS). The most significant differences were observed between HF and hybrid-treated HFC and HFCX flours; a significant decrease in DoS was observed from 24.3 BU to 7.7 BU and 5.7 BU, respectively. DoS12 values were higher by more than double if measured at 12 min of the test for F and with enzymes added (FC or FCX), but 3.5–4 times higher in TF, TFC and TFCX samples, compared to DoS values. Hydrothermal treatment resulted in a significant increase in flour stability to 18.4 min, without significant variability when enzymes were used, but as a result of extended development time. The presence of cellulase or cellulase–xylanase enzymes in TFC and TFCX, as well as in HFC and HFCX samples, increased QN as compared to native and treated flour without enzymes.

Gómez et al. [55] reported that extruded wheat bran addition (2.5 to 20%) increased. DT. Tayefe et al. [56] added hydrothermally treated rice bran to wheat dough which changed the starch present in the bran into a pregelatinized form, hence improving water molecule retention and, when added to the wheat dough, increasing its water absorption capacity, elongating dough development time but lowering dough stability by lowering the gluten content. Tao et al. [57] found increased water absorption of wheat starch extruded at a temperature of 50–70 °C; the addition of 15% of extruded starch decreased DT and strengthened wheat dough consistency.

The modified flours were tested via the Alveograph procedure, which involves measuring the resistance of a dough sample prepared from flour and sodium chloride solution while blowing it evenly. The results of the Alveograph tests are presented in Table 9.

**Table 9:** Alveograph properties of untreated and hybrid-treated flours.

Sample	P (mm)	L (mm)	W (10^−4^ J)	P/L (-)	Ie (%)	SH (-)
F	111 ± 3 ^b^	77 ± 6 ^c,d^	273 ± 18 ^d^	1.45 ± 0.10 ^a^	49.27 ± 0.81 ^c^	1.66 ± 0.02 ^c^
FC	105 ± 1 ^b,c,d^	74 ± 6 ^c^	253 ± 12 ^c,d^	1.42 ± 0.12 ^a^	48.87 ± 0.46 ^c^	1.67 ± 0.03 ^c^
FCX	107 ± 1 ^c,d^	78 ± 3 ^c,d^	266 ± 8 ^c,d^	1.37 ± 0.03 ^a^	49.60 ± 0.61 ^c^	1.66 ± 0.02 ^c^
TF	82 ± 3 ^a^	87 ± 1 ^d^	199 ± 6 ^b^	0.95 ± 0.04 ^a^	44.83 ± 0.29 ^a^	1.48 ± 0.05 ^a^
TFC	98 ± 2 ^b,c^	86 ± 3 ^d^	243 ± 10 ^c,d^	1.12 ± 0.02 ^a^	46.50 ± 0.70 ^b^	1.54 ± 0.02 ^a,b^
TFCX	101 ± 5 ^b,c^	84 ± 3 ^c,d^	249 ± 16 ^c,d^	1.21 ± 0.03 ^a^	46.47 ± 0.35 ^b^	1.55 ± 0.01 ^a,b^
HF	140 ± 6 ^e^	38 ± 1 ^b^	227 ± 13 ^c^	3.71 ± 0.21 ^b^	ND	2.08 ± 0.02 ^e^
HFC	97 ± 3 ^b^	26 ± 7 ^a^	134 ± 31 ^a^	3.97 ± 0.96 ^b,c^	ND	1.84 ± 0.03 ^d^
HFCX	147 ± 4 ^e^	31 ± 1 ^a,b^	194 ± 7 ^b^	4.70 ± 0.19 ^c^	ND	1.61 ± 0.06 ^b,c^

F—flour; T—dry thermal treatment; H—hydrothermal treatment; C—cellulase enzyme; X—xylanase enzyme; P—dough tenacity; L—extensibility; W—baking strength; P/L—configuration ratio; Ie—elasticity index; SH—strain hardening index; ND—no data;^a–e^—means indicated with similar letters in columns do not differ significantly at α = 0.05.

The tested base flour F was characterized by good baking properties, with a baking strength value W of 273 × 10^−4^ J. The addition of the cellulase FC or cellulase–xylanase complex FCX slightly reduced the elasticity and increased the extensibility of the dough, maintaining the elasticity index Ie and the strain hardening index SH at a similar level. The use of thermal treatment significantly reduced the baking value of TF flour, mainly by reducing the elasticity of the dough (P); it also worsened the Ie and SH parameters, from 49.27% (F) to 44.83% (TF) and from 1.66 (F) to 1.48 (TF), respectively. The use of hybrid modification through the incorporation of baking enzymes in the mixture, especially TFCX, allowed for obtaining quality close to that of the F, FC and FCX samples, especially in dough elasticity (P). Dry thermal and hybrid treatment increased the dough extensibility L, but slightly lowered baking strength W, elasticity index Ie and strain hardening SH, with a lesser difference if TFC and TFCX samples were tested (Table 9).

We noted that the applied steam-assisted hydrothermal treatment caused significant changes in the conformation of gluten proteins, preventing proper development of the gluten network or destruction of gluten. This was due either to the steam’s high temperature or by the integrated thermal–enzymatic destruction of the gluten network, which became brittle and short. As a result, the dough showed very poor dough extensibility L (more than a double lowering than that of native F) and increased SH, especially in HF and HFC samples. The P/L index was similar for native F and enzyme-added samples FC and FCX, and slightly lower values were noted in thermally treated flours without/with enzymes. The greater differences were found in P/L with more than triple higher results in the testing of dough made of HF, HFC and HFCX flours. Elasticity index Ie was not possible to obtain in the hydrothermally modified samples. Although the SH coefficient increased to higher levels than in the base flour, this was due to an increase in dough stiffness rather than an actual improvement in flour performance.

Some important correlations were found between rheological properties tested with various methods. Alveograph features were correlated with dough quality from Farinograph tests, with a significant positive correlation at *p* < 0.05 of *p* values with dough stability (0.760) and negatively with DoS12 (−0.928). L results were negatively significantly correlated at *p* < 0.05 with S (−0.946), but positively with DoS12 (0.920). Moreover, baking strength W was negatively correlated with DT (−0.830). P/L was strongly (at *p* < 0.05) correlated with dough stability (0.935) and with DoS12 (−0.923). There were also some significant (at *p* < 0.05) correlations found between dough tenacity P and I-arabinose (−0.627), I-xylose (−0.709) and I-AX (−0.711), which can explain the effect of insoluble fractions of non-starch polysaccharides on dough properties. Soluble fractions of polysaccharides were also found to be related to P, and significant correlations were found for S-xylose (0.828), S-NSP (0.856) and S-AX (0.792) at *p* < 0.05.

For the extrusion treatment and partly for the hydrothermal treatment, it was not possible to assess the rheological properties of the gluten matrix (Ie values) due to the starch gelatinization and denaturation of the gluten protein. All the extruded flours were unable to successfully form a dough when used alone. In wheat dough fortified with extruded bran, Gómez et al. [55] observed changes in P, L and W because of the interrupted gluten–starch matrix and a negative effect on gas retention, resulting in a dough extensibility reduction and tenacity increase associated with poor handling characteristics of doughs. Jødal and Larsen [35] indicated high P/L as a resistant and non-stretchable dough, while low P/L revealed a weak and stretchy dough.

### 3.6. Treatment Effect on X-ray-Detected Structure

**Figure 2:**
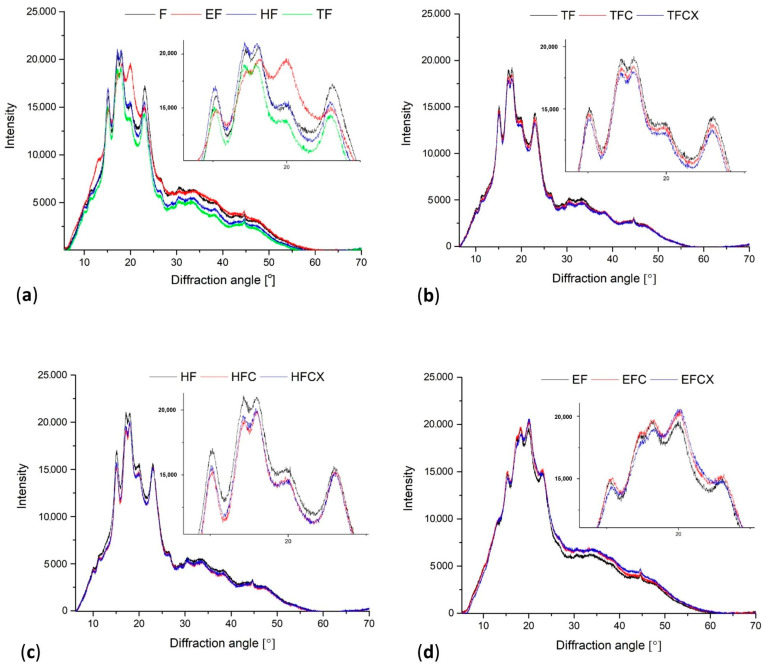
X-ray diffraction patterns of modified wheat flour: (**a**) comparison of various treatments without enzymes, (**b**) effect of enzymes in dry thermal treatment, (**c**) effect of enzymes in hydrothermal and (**d**) effect of enzymes in extrusion treatment.

The treated flour X-ray diffractometry profiles are shown in Figure 2. Zeng et al. [58] consider the X-ray diffraction pattern as the “fingerprint” of plant starch crystallinity. Regarding X-ray diffraction lines, the crystal structure of starch can be divided into four types, where A, B and C types are the natural crystal structures of starch, and the structure V type is typical for complexes created by amylose and lipids.

The wheat starches present standard A-type crystallinity diffraction patterns at 2θ with peaks near 15°, a strong doublet around 2θ ≈ 17° and 18° and a third main reflection around 23° [45]. The peak at 2θ ≈ 20° is the amorphous peak of amylose and lipids, and a higher peak intensity suggests its higher content [59]. In wheat, depending on the variety, if the relative intensity of the peak at 23° is higher, then the 15° peak intensity is generally lower. The presence of peaks at certain angles differs depending on wheat flour treatment conditions and enzyme presence. In native, thermal and hydrothermal modified samples, all characteristic patterns were found to be at similar diffraction angles: at 15°, with a strong doublet at 17 and 18°, and at 23°. In wheat flour treated via steam (HF), however, X-ray patterns showed sharper peaks in comparison to that of the other treatments (Figure 2a), suggesting the prevalence of A-type starch [58]. Dry thermal treatment in TF samples induced relocation of the highest peak point to 20°, indicating that these samples contain the lowest amount of amorphous structures. Regarding the extruded flour, the peak intensity at 20° was the highest, suggesting the presence of amorphous phases of amylose and lipids; however, the 17° peak was the lowest. In TF, the peak intensity was the lowest at 20° and 22°, and in TF and EF, significant decreases in peak intensity at 15 and 17° were observed.

Figure 2b–d reveal the pattern differences in modified flours without/with added enzymes. Differences were slight in dry-thermal-treated samples (Figure 2b), but in TFC and TFCX, the heights of all peak intensities were lower. A similar but stronger trend was seen in HFC and HFCX, with a more significant decrease in peak intensities, as compared to enzyme-free HF (Figure 2c). The extruded samples EFC and EFCX, which contain enzymes (Figure 2d), showed higher peak positions at 20°, as compared to EF samples. CX complex addition, however, lowered the intensity of the peaks at 15° and at 17° and 18°, suggesting a reorganization of A-type crystallinity. Indeed, Li et al. [45] found that amylose content was significantly positively correlated with the intensity of the diffraction peak at 23° and the crystallinity of diffraction peaks at 17–18°. The introduction of enzymes into hybrid enzymatic–extrusion treatments also changed diffraction intensity at the range of 25 to 45° by increasing the surface area under the baselined curves, as compared to EF, suggesting more amorphous structures in the EFC and EFCX samples, as compared to other treatment methods (Figure 2a). Tao et al. [57] identified in native wheat starch the presence of all four A-type patterns, and after extrusion, the crystalline peaks were less pronounced, especially at 17, 18 and 23°. The loss of crystallinity was brought about because high extrusion temperature caused thermal degradation of the starch amylopectin fraction at its branching points. Liu et al. [52] used a single-screw extrusion treatment to modify rice starch and reported a gradual weakening of all peaks associated with A-type crystallinity patterns after extrusion. These became more amorphous as the initial moisture content increased in the extruded starch. They found a clear crystallinity peak at 20° for extruded starch (not present in native rice starch), indicating V-type crystallinity that can be associated with the formation of the amylose–lipid complex. Merayo et al. [60] reported changes in a peak located at 20° (2θ scale), indicating the formation of amylose–lipid complexes in their investigation of the extrusion of red and yellow corn flours during spaghetti production. He et al. [61] tested cellulose nanofiber/polyaniline film composites and found two peaks at 2θ = 15.24° and 22.4° for cellulose-based film (this is the classic cellulose I structure). These peaks were superimposed on broad scatterings placed between 15° and 25°, which were ascribed to the periodicity parallel and perpendicular to the polymer chains of polymerized composite film, respectively.

### 3.7. Microstructure of Native and Processed Flours

**Figure 3:**
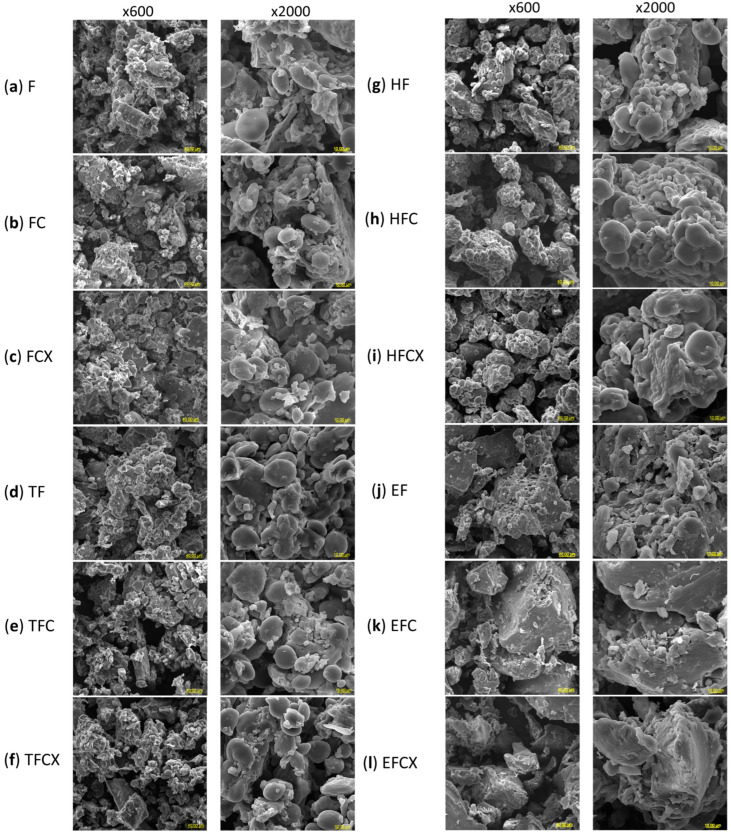
SEM structure of modified wheat flour at various magnifications (600× and 2000×): (**a**) native flour, (**b**) native flour with cellulase, (**c**) native flour with cellulase–xylanase complex, (**d**) dry-treated flour, (**e**) dry-treated flour with cellulase, (**f**) dry-treated flour with cellulase–xylanase complex, (**g**) hydrothermally treated flour, (**h**) hydrothermally treated flour with cellulase, (**i**) hydrothermally treated flour with cellulase–xylanase complex, (**j**) extruded flour, (**k**) extruded flour with cellulase and (**l**) extruded flour with cellulase–xylanase complex.

The microstructure of native and treated wheat flours is presented in Figure 3. SEM pictures were taken with low (600×) and high (2000×) magnifications. Native flour (Figure 3a) displayed the presence of various fractions visible as large and small starch granules, parts of fibrous components coming from bran and elongated structures coming from protein. The diverse wheat starch granule sizes confirmed the presence of both granule types: A-type (diameter over 9.9 μm) and B-type (diameter below 9.9 μm). In native wheat flour, A-type granules contain up to 70% of the volume and 10% of the total number of starch granules, and B-type granules contain approximately 30% of the volume and 90% of the total number of granules [62]. Very fine (<2.0 μm) C-type starch granules have also been reported, although this type of granule may also represent a B-type granule [59]. A-type and B-type starch granules show different morphologies, wherein the A-type has a disk-like shape with possible grooves or indentations, and the B-type exhibits spherical, ellipsoidal, angular and irregular shapes, and is tightly packed within the endosperm [49].

In a native flour, after the enzyme addition, slight agglomeration may be observed in FC and FCX flours (Figure 3b,c, respectively). This may be a result of enzyme activity and the initiation of hydrolysis of the linear fractions of polysaccharides, especially cellulose and hemicellulose, visible as groups of glued flour particles with both A- and B-type starch placed close to each other. Enzyme addition to low-moisture flour results in incomplete hydrolysis, but the structure of wheat flour can change, e.g., an increase in S-NSP content, especially S-AX, can thus increase hydration possibilities and DT, but lower viscosity, breakdown and setback.

After dry thermal treatment, more singular starch granules of larger dimensions are noticeable, suggesting the presence of heated and swollen starch granules placed loosely in the TF flour (Figure 3d). In TF, increased content of I-AX and I-NSP and of T-AX and T-NSP, lower viscosity and hydration were noted, as compared to F, but the addition of enzymes generated an opposite effect in TFC and TFCX samples, especially when the cellulase–xylanase complex was incorporated. In these samples, we noted finer particle packing (Figure 3e,f) with empty space between starch granules, which allowed for more solvent absorption and increased DT and dough extensibility. The lower moisture of dry heated flours might, however, contribute to this effect.

In steam-treated flours (the HF sample), we observed a visible partial agglomeration (Figure 3g) that was more intensive with enzyme incorporation (Figure 3h,i). Formation of these agglomerates with swollen and partly gelatinized starch granules and with the presence of more finer granules of lower dimensions sufficiently decreased hydration ability, shortened dough extensibility and lowered baking strength with increasing maximum viscosity.

Small B-type granules are characterized by greater resistance to hydrolysis and exhibit lower gelatinization temperatures than the A-type structure that was observed after HF and EF treatments. The most significant changes were observed in the extruded flour, without (Figure 3j) and with enzyme addition (Figure 3k,l). According to Bouasla et al. [37], raw flour is molten inside an extruder and gelatinized in the extended range. We confirmed this by seeing the melted and compact internal structure of the starch–protein–lipid matrix with large clusters formed due to association after treatment in the presence of water (27%) and the absence of free starch granules, especially in the EFC and EFCX samples (Figure 3k,l). A similar conclusion was found by Wu et al. [11]; they showed that the extruder action caused the starch to decompose. In the extruded flours, the lowered I-NSP and increased S-AX in this melted internal structure significantly enhanced the flour’s hydration properties, but decreased dough stability, GPI, C3, C4 and C5—and made dough creation impossible. Cervantes-Ramírez et al. [63] observed an integrated amorphous matrix of corn starch formed as the effect of extrusion treatment. However, some granules remained visible due to the fatty acids acting as a protective (lubricating) coating during extrusion and significantly reducing physical damage of starch granules.

## 4. Conclusions

Wheat flour blend modification had variable effects on composition, rheology and structure depending on treatment conditions and enzyme applications. The results confirmed that hybrid treatments incorporating cellulase and/or cellulase–xylanase complex enzymes modified polysaccharide compositions and techno-functional properties. The thermal treatment turned out to have the least destructive effect on the protein quality, especially if the cellulase–xylanase enzyme complex was incorporated. The dough matrix, however, became more resistant to mixing because of protein structure improvement. Intensive treatment in hydrothermal and extrusion methods had a negative impact on the quality of protein fractions but significantly changed gelling properties whether performed without/with enzymes. Incorporating the cellulase–xylanase enzyme complex resulted in a significant increase in the soluble fractions of arabinoxylans, which have a structure-forming function in the dough matrix and participate in water management. Significant changes were observed in the structure and microstructure of the modified flours, especially when extrusion was applied. The techno-functional properties of modified flours, especially their hydration and pasting properties, as well as dough rheology, may be developed by proper treatment methods for their use in various applications. Extruded flours with high water absorption may be an interesting alternative to pregelatinized/modified starch or hydrocolloids in the bakery production process with low dosing and a significant effect in increasing the bread yield. Furthermore, these types of flours do not need to be labeled as additives but as wheat flour, which will facilitate clear labeling—currently the preferred trend in the food industry. Further investigation will include using the obtained modified flours as clean-label ingredients in wheat bread in order to verify the technological and quality features obtained during processing in the final bakery products.

## Figures and Tables

**Table 1:** Selected chemical properties of untreated and hybrid-treated flours.

Sample	Moisture (%)	Protein (%)	Fat (%)	Ash (%)	IDF (%)	SDF (%)	TDF (%)	IDF/ SDF (-)
F	13.93 0.08 ^e^	14.62 ± 0.06 ^a,b^	1.31 ± 0.01 ^d^	0.72 ± 0.02 ^a^	3.94 ± 0.04 ^d^	2.86 ± 0.01 ^e^	6.80 ± 0.03 ^e^	1.38
FC	13.89 ± 0.09 ^e^	14.52 ± 0.03 ^a^	1.39 ± 0.02 ^f^	0.74 ± 0.02 ^a,b,c,d^	3.81 ± 0.03 ^c^	2.33 ± 0.03 ^b^	6.14 ± 0.05 ^c^	1.63
FCX	13.76 ± 0.07 ^e^	14.64 ± 0.01 ^a,b,c^	1.30 ± 0.01 ^d^	0.73 ± 0.01 ^a,b^	4.10 ± 0.03 ^e^	2.56 ± 0.01 ^c^	6.66 ± 006 ^d^	1.61
TF	7.01 ± 0.06 ^a^	14.75 ± 0.05 ^c,d^	1.34 ± 0.03 ^d,e^	074 ± 0.01 ^a,b,c^	4.70 ± 0.02 ^h^	2.87 ± 0.02 ^e^	7.57 ± 0.01 ^h^	1.64
TFC	7.17 ± 0.13 ^a^	14.92 ± 0.04 ^e,f^	1.38 ± 0.01 ^e,f^	0.76 ± 0.01 ^b,c,d,e^	4.25 ± 0.03 ^f^	2.79 ± 0.01 ^d^	7.04 ± 0.04 ^f,g^	1.52
TFCX	7.23 ± 0.11 ^a^	15.11 ± 0.05 ^g^	1.36 ± 0.02 ^e,f^	0.82 ± 0.02 ^f^	4.22 ± 0.01 ^f^	2.74 ± 0.03 ^d^	6.96 ± 0.03 ^f^	1.54
HF	8.98 ± 0.09 ^c^	14.85 ± 0.03 ^d,e^	1.37 ± 0.02 ^e,f^	0.76 ± 0.02 ^b,c,d,e^	3.91 ± 0.04 ^d^	3.12 ± 0.01 ^g^	7.02 ± 0.07 ^f,g^	1.25
HFC	9.17 ± 0.12 ^c,d^	14.81 ± 0.04 ^d,e^	1.30 ± 0.01 ^d^	0.78 ± 0.01 ^d,e^	4.60 ± 0.03 ^g^	2.89 ± 0.01 ^e,f^	7.49 ± 0.03 ^h^	1.59
HFCX	9.28 ± 0.06 ^d^	15.02 ± 0.06 ^f,g^	1.30 ± 0.02 ^d^	0.73 ± 0.02 ^a,b^	4.05 ± 0.04 ^e^	2.55 ± 0.03 ^c^	6.60 ± 0.06 ^d^	1.58
EF	8.18 ± 0.06 ^b^	14.76 ± 0.04 ^c,d^	0.22 ± 0.02 ^b^	0.77 ± 0.01 ^c,d,e^	3.05 ± 0.03 ^a^	2.24 ± 0.02 ^a^	5.29 ± 0.05 ^a^	1.37
EFC	8.06 ± 0.08 ^b^	14.67 ± 0.05 ^b,c^	0.16 ± 0.02 ^a^	0.78 ± 0.01 ^e^	4.20 ± 0.03 ^f^	2.95 ± 0.03 ^f^	7.15 ± 0.06 ^g^	1.42
EFCX	8.26 ± 0.13 ^b^	14.89 ± 0.05 ^e,f^	0.29 ± 0.02 ^c^	0.83 ± 0.01 ^f^	3.15 ± 0.02 ^b^	2.57 ± 0.02 ^c^	5.73 ± 0.03 ^b^	1.23

F—flour; T—dry thermal treatment; H—hydrothermal treatment; E—extrusion treatment; C—cellulase enzyme; X—xylanase enzyme; TDF—total dietary fiber; IDF—insoluble dietary fiber; SDF—soluble dietary fiber; ^a–h^—means indicated with similar letters in columns do not differ significantly at α = 0.05.

**Table 2:** Insoluble non-starch polysaccharide and arabinoxylan content in untreated and hybrid-treated flours.

Sample	Insoluble Polysaccharides					
	I-Mannose (%)	I-Galactose (%)	I-Glucose (%)	I-Arabinose (%)	I-Xylose (%)	I-A/X (-)
F	0.131 ± 0.002 ^a,b^	0.078 ± 0.003 ^a,b,c^	0.548 ± 0.024 ^b,c^	0.549 ± 0.019 ^d^	0.759 ± 0.019 ^e,f^	0.723 ± 0.007 ^a^
FC	0.133 ± 0.005 ^a,b^	0.074 ± 0.002 ^a,b^	0.535 ± 0.005 ^b,c^	0.458 ± 0.027 ^b,c^	0.674 ± 0.004 ^c,d^	0.680 ± 0.044 ^a^
FCX	0.142 ± 0.017 ^a,b^	0.081 ± 0.007 ^a,b,c^	0.528 ± 0.003 ^a,b,c^	0.518 ± 0.020 ^b,c,d^	0.651 ± 0.033 ^b,c^	0.798 ± 0.072 ^a^
TF	0.137 ± 0.016 ^a,b^	0.097 ± 0.008 ^b,c^	0.662 ± 0.018 ^e^	0.582 ± 0.009 ^d^	0.842 ± 0.024 ^g^	0.692 ± 0.009 ^a^
TFC	0.129 ± 0.009 ^a,b^	0.081 ± 0.005 ^a,b,c^	0.538 ± 0.012 ^b,c^	0.536 ± 0.013 ^c,d^	0.735 ± 0.010 ^d,e,f^	0.729 ± 0.027 ^a^
TFCX	0.168 ± 0.013 ^b^	0.072 ± 0.013 ^a,b^	0.630 ± 0.015 ^d,e^	0.498 ± 0.031 ^b,c,d^	0.691 ± 0.019 ^c,d,e^	0.721 ± 0.025 ^a^
HF	0.154 ± 0.015 ^a,b^	0.087 ± 0.009 ^a,b,c^	0.654 ± 0.001 ^e^	0.503 ± 0.004 ^b,c,d^	0.695 ± 0.007 ^c,d,e^	0.723 ± 0.013 ^a^
HFC	0.231 ± 0.031 ^c^	0.100 ± 0.001 ^c^	0.657 ± 0.001 ^e^	0.539 ± 0.040 ^c,d^	0.787 ± 0.002 ^f,g^	0.685 ± 0.052 ^a^
HFCX	0.153 ± 0.006 ^a,b^	0.081 ± 0.004 ^a,b,c^	0.569 ± 0.012 ^c^	0.446 ± 0.009 ^b^	0.589 ± 0.022 ^b^	0.758 ± 0.013 ^a^
EF	0.120 ± 0.005 ^a^	0.074 ± 0.001 ^a,b^	0.573 ± 0.003 ^c,d^	0.507 ± 0.007 ^b,c,d^	0.694 ± 0.014 ^c,d,e^	0.731 ± 0.005 ^a^
EFC	0.131 ± 0.014 ^a,b^	0.069 ± 0.014 ^a^	0.469 ± 0.052 ^a^	0.516 ± 0.078 ^b,c,d^	0.642 ± 0.043 ^b,c^	0.800 ± 0.068 ^a^
EFCX	0.129 ± 0.003 ^a,b^	0.091 ± 0.016 ^a,b,c^	0.496 ± 0.028 ^a,b^	0.353 ± 0.013 ^a^	0.474 ± 0.057 ^a^	0.749 ± 0.063 ^a^

F—flour; T—dry thermal treatment; H—hydrothermal treatment; E—extrusion treatment; C—cellulase enzyme; X—xylanase enzyme; I-A/X—insoluble arabinose-to-xylose ratio; ^a–g^—means indicated with similar letters in columns do not differ significantly at α = 0.05.

**Table 3:** Soluble non-starch polysaccharide and arabinoxylan content in untreated and hybrid-treated flours.

Sample	Soluble Polysaccharides					
	S-Mannose (%)	S-Galactose (%)	S-Glucose (%)	S-Arabinose (%)	S-Xylose (%)	S-A/X (-)
F	0.265 ± 0.008 ^b,c^	0.118 ± 0.000 ^a^	0.352 ± 0.017 ^a,b,c,d^	0.276 ± 0.008 ^a,b^	0.328 ± 0.012 ^b^	0.842 ± 0.008 ^c,d^
FC	0.275 ± 0.001 ^c,d^	0.123 ± 0.003 ^a^	0.313 ± 0.012 ^a^	0.281 ± 0.002 ^a,b^	0.335 ± 0.003 ^b^	0.839 ± 0.000 ^c,d^
FCX	0.274 ± 0.006 ^c,d^	0.124 ± 0.002 ^a^	0.381 ± 0.005 ^d,e^	0.287 ± 0.001 ^a,b^	0.351 ± 0.007 ^b^	0.819 ± 0.013 ^b,c,d^
TF	0.239 ± 0.014 ^a,b^	0.121 ± 0.00 ^a^	0.356 ± 0.004 ^b,c,d^	0.296 ± 0.002 ^b^	0.324 ± 0.022 ^b^	0.916 ± 0.069 ^d^
TFC	0.242 ± 0.012 ^a,b^	0.131 ± 0.005 ^a^	0.382 ± 0.002 ^d,e^	0.263 ± 0.006 ^a^	0.322 ± 0.009 ^b^	0.818 ± 0.002 ^a,b,c,d^
TFCX	0.249 ± 0.013 ^a,b,c^	0.115 ± 0.004 ^a^	0.324 ± 0.025 ^a,b^	0.271 ± 0.004 ^a,b^	0.342 ± 0.004 ^b^	0.791 ± 0.002 ^a,b,c^
HF	0.295 ± 0.005 ^d^	0.131 ± 0.002 ^a^	0.392 ± 0.008 ^d,e^	0.275 ± 0.017 ^a,b^	0.396 ± 0.024 ^c^	0.698 ± 0.085 ^a^
HFC	0.227 ± 0.007 ^a^	0.118 ± 0.013 ^a^	0.420 ± 0.024 ^e^	0.281 ± 0.012 ^a,b^	0.268 ± 0.011 ^a^	1.048 ± 0.00 ^e^
HFCX	0.241 ± 0.008 ^a,b^	0.121 ± 0.011 ^a^	0.415 ± 0.004 ^e^	0.360 ± 0.001 ^c^	0.468 ± 0.009 ^d^	0.769 ± 0.018 ^a,b,c^
EF	0.257 ± 0.001 ^b,c^	0.119 ± 0.000 ^a^	0.322 ± 0.000 ^a,b^	0.268 ± 0.008 ^a,b^	0.341 ± 0.007 ^b^	0.788 ± 0.005 ^a,b,c^
EFC	0.300 ± 0.020 ^d^	0.121 ± 0.013 ^a^	0.369 ± 0.025 ^c,d^	0.283 ± 0.025 ^a,b^	0.401 ± 0.013 ^c^	0.708 ± 0.087 ^a,b^
EFCX	0.262 ± 0.006 ^b,c^	0.104 ± 0.025 ^a^	0.328 ± 0.008 ^a,b^	0.418 ± 0.005 ^d^	0.590 ± 0.010 ^e^	0.709 ± 0.002 ^a,b^

F—flour; T—dry thermal treatment; H—hydrothermal treatment; E—extrusion treatment; C—cellulase enzyme; X—xylanase enzyme; S-A/X—soluble arabinose-to-xylose ratio; ^a–e^—means indicated with similar letters in columns do not differ significantly at α = 0.05.

**Table 4:** Non-starch polysaccharide and arabinoxylan content in untreated and hybrid-treated flours.

Sample	Polysaccharide Fractions					
	I-AX (%)	S-AX (%)	T-AX (%)	I-NSP (%)	S-NSP (%)	T-NSP (%)
F	1.31 ± 0.04 ^d,e,f^	0.604 ± 0.020 ^b,c^	1.912 ± 0.058 ^b,c^	2.064 ± 0.009 ^d,e^	1.340 ± 0.004 ^a^	3.404 ± 0.004 ^b,c^
FC	1.13 ± 0.02 ^b,c^	0.616 ± 0.005 ^b,c^	1.749 ± 0.018 ^a^	1.875 ± 0.025 ^b,c^	1.327 ± 0.012 ^a^	3.202 ± 0.037 ^a^
FCX	1.17 ± 0.01 ^b,c,d^	0.638 ± 0.009 ^c,d^	1.807 ± 0.004 ^a,b^	1.921 ± 0.008 ^b,c,d^	1.416 ± 0.001 ^b^	3.337 ± 0.007 ^a,b,c^
TF	1.42 ± 0.03 ^f^	0.620 ± 0.020 ^b,c^	2.044 ± 0.013 ^c^	2.319 ± 0.074 ^f^	1.335 ± 0.037 ^a^	3.654 ± 0.037 ^e^
TFC	1.27 ± 0.00 ^c,d,e^	0.585 ± 0.015 ^a,b^	1.856 ± 0.012 ^a,b^	2.020 ± 0011 ^c,d,e^	1.340 ± 0.004 ^a^	3.360 ± 0.015 ^a,b,c^
TFCX	1.19 ± 0.05 ^c,d,e^	0.613 ± 0.007 ^b,c^	1.802 ± 0.042 ^a,b^	2.059 ± 0.064 ^d,e^	1.301 ± 0.035 ^a^	3.361 ± 0.099 ^a,b,c^
HF	1.20 ± 0.00 ^c,d,e^	0.671 ± 0.007 ^d,e^	1.869 ± 0.010 ^a,b^	2.093 ± 0.027 ^e^	1.489 ± 0.018 ^c^	3.581 ± 0.045 ^d,e^
HFC	1.33 ± 0.04 ^e,f^	0.548 ± 0.023 ^a^	1.875 ± 0.016 ^a,b^	2.314 ± 0.069 ^f^	1.313 ± 0.027 ^a^	3.627 ± 0.042 ^e^
HFCX	1.04 ± 0.03 ^b^	0.828 ± 0.008 ^f^	1.863 ± 0.023 ^a,b^	1.838 ± 0.018 ^b^	1.605 ± 0.015 ^d^	3.443 ± 0.003 ^d^
EF	1.20 ± 0.02 ^c,d,e^	0.609 ± 0.015 ^b,c^	1.810 ± 0.036 ^a,b^	1.967 ± 0.014 ^b,c,d,e^	1.307 ± 0.016 ^a^	3.274 ± 0.030 ^a,b^
EFC	1.16 ± 0.12 ^b,c^	0.684 ± 0.012 ^e^	1.842 ± 0.134 ^a,b^	1.828 ± 0.041 ^b^	1.473 ± 0.021 ^b,c^	3.302 ± 0.062 ^a,b,c^
EFCX	0.83 ± 0.07 ^a^	1.009 ± 0.015 ^g^	1.836 ± 0.055 ^a,b^	1.543 ± 0.111 ^a^	1.703 ± 0.012 ^e^	3.246 ± 0.123 ^a,b^

F—flour; T—dry thermal treatment; H—hydrothermal treatment; E—extrusion treatment; C—cellulase enzyme; X—xylanase enzyme; I-AX—insoluble arabinoxylans; S-AX—soluble arabinoxylans; T-AX—total arabinoxylans; I-NSPs—insoluble non-starch polysaccharides; S-NSPs—soluble non-starch polysaccharides; T-NSPs—total non-starch polysaccharides; ^a–g^—means indicated with similar letters in columns do not differ significantly at α = 0.05.

**Table 5:** SRC values of untreated and hybrid-treated flours.

Sample	SRCWa (%)	SRCSu (%)	SRCLa (%)	SRCSc (%)	GPI (-)
F	70.005 ± 0.062 ^d^	114.973 ± 0.770 ^c,d^	118.294 ± 0.595 ^c,d^	87.839 ± 0.256 ^b,c^	0.583 ± 0.005 ^c^
FC	70.427 ± 0.049 ^d^	116.916 ± 1.228 ^d^	118.289 ± 0.624 ^c,d^	86.971 ± 0.080 ^b,c^	0.580 ± 0.001 ^b,c^
FCX	75.496 ± 0.278 ^e^	119.827 ± 0.876 ^e^	126.504 ± 0.950 ^e^	88.702 ± 0.153 ^c^	0.607 ± 0.003 ^d^
TF	66.757 ± 0.171 ^b^	113.042 ± 0.921 ^c^	112.155 ± 0.959 ^b^	85.038 ± 0.233 ^b^	0.566 ± 0.002 ^b^
TFC	68.594 ± 0.097 ^c^	115.545 ± 0.407 ^d^	115.279 ± 1.645 ^b,c^	87.596 ± 0.073 ^b,c^	0.567 ± 0.007 ^b,c^
TFCX	70.049 ± 0.820 ^d^	119.550 ± 1.698 ^e^	120.105 ± 1.256 ^d^	87.912 ± 0.360 ^b,c^	0.579 ± 0.011 ^b,c^
HF	64.765 ± 0.292 ^a^	100.706 ± 0.493 ^b^	102.786 ± 1.578 ^a^	81.098 ± 0.066 ^a^	0.565 ± 0.008 ^b^
HFC	66.656 ± 0.169 ^b^	96.238 ± 0.761 ^a^	102.717 ± 0.601 ^a^	81.218 ± 0.424 ^a^	0.579 ± 0.001 ^b,c^
HFCX	66.324 ± 0.146 ^b^	99.793 ± 0.774 ^b^	105.804 ± 1.943 ^a^	81.667 ± 0.422 ^a^	0.583 ± 0.010 ^c^
EF	167.158 ± 0.227 ^f^	161.398 ± 0.224 ^f^	172.324 ± 0.952 ^f^	235.073 ± 1.585 ^d^	0.435 ± 0.001 ^a^
EFC	177.957 ± 0.609 ^g^	166.261 ± 0.363 ^g^	183.706 ± 1.429 ^g^	248.603 ± 2.871 ^e^	0.443 ± 0.001 ^a^
EFCX	216.286 ± 0.156 ^h^	184.618 ± 0.087 ^h^	210.365 ± 0.588 ^h^	295.415 ± 0.440 ^f^	0.438 ± 0.001 ^a^

F—flour; T—dry thermal treatment; H—hydrothermal treatment; E—extrusion treatment; C—cellulase enzyme; X—xylanase enzyme; SRC—Solvent Retention Capacity; Wa—distilled water; Su—50% sucrose-in-water solution; La—5% lactic acid-in-water solution; Sc—5% sodium carbonate-in-water solution; GPI—gluten performance index; ^a–h^—means indicated with similar letters in columns do not differ significantly at α = 0.05.

**Table 6:** Pasting properties of untreated and hybrid-treated flours.

Sample	Maximum Viscosity (mPas)	Through Viscosity (mPas)	Final Viscosity (mPas)	Breakdown (mPas)	Setback (mPas)	Beginning of Gelatinization (°C)	End of Gelatinization (°C)
F	1564 ± 9 ^h^	436 ± 3 ^i^	1225 ± 2 ^g^	1128 ± 6 ^g^	789 ± 4 ^h^	60.2 ± 0.0 ^c^	86.6 ± 0.1 ^i^
FC	1313 ± 4 ^e^	322 ± 1 ^e^	935 ± 3 ^e^	990 ± 3 ^e^	612 ± 2 ^e^	60.5 ± 0.0 ^c^	85.5 ± 0.0 ^f^
FCX	1204 ± 3 ^d^	281 ± 3 ^d^	783 ± 9 ^c^	923 ± 4 ^d^	502 ± 7 ^c^	60.2 ± 0.1 ^c^	85.2 ± 0.1 ^e^
TF	1014 ± 15 ^a^	194 ± 3 ^a^	496 ± 12 ^a^	820 ± 12 ^b^	302 ± 9 ^a^	60.3 ± 0.1 ^c^	83.2 ± 0.1 ^a^
TFC	1108 ± 17 ^b^	240 ± 2 ^b^	643 ± 2 ^b^	867 ± 16 ^c^	402 ± 1 ^b^	60.3 ± 0.1 ^c^	83.9 ± 0.1 ^b^
TFCX	1148 ± 10 ^c^	256 ± 4 ^c^	661 ± 7 ^b^	901 ± 2 ^c,d^	404 ± 2 ^b^	60.0 ± 0.1 ^c^	84.3 ± 0.1 ^c^
HF	1432 ± 26 ^g^	351 ± 3 ^f^	892 ± 9 ^d^	1073 ± 29 ^f^	540 ± 9 ^d^	60.2 ± 0.1 ^c^	84.6 ± 0.1 ^d^
HFC	1975 ± 7 ^j^	623 ± 1 ^l^	1519 ± 6 ^j^	1353 ± 7 ^i^	896 ± 4 ^j^	60.3 ± 0.1 ^c^	85.8 ± 0.1 ^g^
HFCX	1785 ± 6 ^i^	533 ± 5 ^k^	1290 ± 12 ^h^	1253 ± 2 ^h^	757 ± 7 ^g^	60.1 ± 0.1 ^c^	85.3 ± 0.1 ^e,f^
EF	1363 ± 4 ^f^	480 ± 1 ^j^	1362 ± 4 ^i^	883 ± 3 ^c^	882 ± 5 ^j^	38.2 ± 0.9 ^b^	86.7 ± 0.2 ^i^
EFC	1215 ± 16 ^d^	405 ± 5 ^h^	1221 ± 10 ^g^	810 ± 12 ^a,b^	816 ± 5 ^i^	37.2 ± 0.8 ^b^	86.3 ± 0.1 ^h^
EFCX	1150 ± 16 ^c^	366 ± 4 ^g^	1092 ± 7 ^f^	781 ± 13 ^a^	726 ± 5 ^f^	35.2 ± 0.6 ^a^	86.2 ± 0.1 ^h^

F—flour; T—dry thermal treatment; H—hydrothermal treatment; E—extrusion treatment; C—cellulase enzyme; X—xylanase enzyme; ^a–l^—means indicated with similar letters in columns do not differ significantly at α = 0.05.

**Table 8:** Farinograph properties of untreated and hybrid-treated flours.

Sample	WA500 (%)	WA 14% (%)	DT (min)	S (min)	DoS (BU)	DoS12 (BU)	QN (-)
F	60.7 ± 0.3 ^a^	59.5 ± 0.3 ^c^	3.1 ± 0.6 ^a^	14.1 ± 0.7 ^c^	21.7 ± 3.2 ^d,e,f^	38.0 ± 2.6 ^c^	118.3 ± 10.3 ^a^
FC	61.0 ± 0.1 ^a,b^	60.2 ± 0.1 ^d^	2.7 ± 0.3 ^a^	12.1 ± 0.9 ^a,b^	29.3 ± 5.0 ^f^	46.3 ± 3.1 ^d,e^	96.3 ± 9.9 ^a^
FCX	61.3 ± 0.0 ^b^	60.5 ± 0.0 ^d,e^	3.8 ± 0.1 ^a,b^	13.1 ± 0.1 ^b,c^	19.7 ± 1.2 ^c,d,e^	42.0 ± 0.0 ^c,d^	128.0 ± 1.7 ^a,b^
TF	66.0 ± 0.2 ^e^	58.5 ± 0.2 ^b^	5.9 ± 0.8 ^b,c^	11.5 ± 0.4 ^a^	17.0 ± 1.0 ^c,d,e^	58.7 ± 2.5 ^f^	126.0 ± 2.6 ^a,b^
TFC	67.9 ± 0.1 ^f^	60.8 ± 0.1 ^e^	6.1 ± 0.9 ^c^	11.5 ± 0.3 ^a^	15.3 ± 3.1 ^b,c,d^	57.7 ± 3.5 ^f^	126.7 ± 5.5 ^a,b^
TFCX	68.2 ± 0.1 ^f^	61.8 ± 0.1 ^f^	6.5 ± 0.9 ^c^	11.5 ± 0.6 ^a^	12.0 ± 2.0 ^a,b,c^	53.3 ± 2.5 ^e,f^	132.7 ± 2.5 ^a,b^
HF	64.6 ± 0.1 ^d^	57.3 ± 0.1 ^a^	2.9 ± 0.1 ^a^	18.4 ± 0.1 ^d^	24.3 ± 4.7 ^e,f^	27.7 ± 3.8 ^b^	161.3 ± 36.3 ^b^
HFC	63.5 ± 0.1 ^c^	58.3 ± 0.1 ^b^	18.5 ± 1.6 ^d^	17.3 ± 0.6 ^d^	7.7 ± 2.1 ^a,b^	ND	200.0 ± 0.0 ^c^
HFCX	63.7 ± 0.1 ^c^	58.5 ± 0.2 ^b^	6.8 ± 0.2 ^c^	18.4 ± 0.1 ^d^	5.7 ± 0.6 ^a^	7.3 ± 1.2 ^a^	200.0 ± 0.0 ^c^

F—flour; T—dry thermal treatment; H—hydrothermal treatment; C—cellulase enzyme; X—xylanase enzyme; WA500—water absorption at 500 BU; WA 14%—water absorption corrected for 14%; DT—dough development time; S—stability; DoS—dough softening in time; DoS12—dough softening after 12 min; QN—quality number; ND—no data; ^a–f^—means indicated with similar letters in columns do not differ significantly at α = 0.05.

## Data Availability

The original contributions presented in the study are included in the article. Further inquiries can be directed to the corresponding author.
